# Family ties: the multilevel effects of households and kinship on the networks of individuals

**DOI:** 10.1098/rsos.172159

**Published:** 2018-04-18

**Authors:** Jeremy Koster

**Affiliations:** Department of Anthropology, University of Cincinnati, Cincinnati, OH 45221-0380, USA

**Keywords:** social network analysis, kin selection, multilevel modelling

## Abstract

Among social mammals, humans uniquely organize themselves into communities of households that are centred around enduring, predominantly monogamous unions of men and women. As a consequence of this social organization, individuals maintain social relationships both within and across households, and potentially there is conflict among household members about which social ties to prioritize or de-emphasize. Extending the logic of structural balance theory, I predict that there will be considerable overlap in the social networks of individual household members, resulting in a pattern of group-level reciprocity. To test this prediction, I advance the Group-Structured Social Relations Model, a generalized linear mixed model that tests for group-level effects in the inter-household social networks of individuals. The empirical data stem from social support interviews conducted in a community of indigenous Nicaraguan horticulturalists, and model results show high group-level reciprocity among households. Although support networks are organized around kinship, covariates that test predictions of kin selection models do not receive strong support, potentially because most kin-directed altruism occurs within households, not between households. In addition, the models show that households with high genetic relatedness in part from children born to adulterous relationships are less likely to assist each other.

## 1. Introduction

Among hominoids, humans are uniquely organized in multifamily communities [1,2]. Cross-culturally, that is, married individuals and their offspring routinely aggregate into larger communities. The ethological uniqueness of this social organization is easily overshadowed by the ethnological diversity of households and their composition, exhibiting such expansive diversity that anthropologists struggle to identify the defining characteristics of human households [3]. Nevertheless, the ubiquitous human pattern is for small groups of related individuals to share domiciles and cooking areas while interacting regularly and peacefully with other comparable households (where ‘households’ is defined broadly to include analogous residence units).

As a consequence of this social structure, individuals maintain social relationships both within and across households. Whereas the intra-household coordination of production and consumption requires careful attention and effort [4], human behavioural ecologists have documented that typically there is also considerable sharing of labour and resources across households [5–9]. These exchanges often conform to predictions of evolutionary models such as kin selection and contingent reciprocity, but especially when commodities are transacted among groups, such as households, there is potential for a ‘targeting problem’ [10]. Food sharing is an apt example. Donors of food may wish to direct resources to a specific individual, but when food is prepared and shared communally within households, consumers of the given food may include unintended and undesirable recipients [11]. The targeting problem extends to conflicts of interest among members of the donating household, as when husbands and wives maintain divergent preferences to share resources with their respective consanguineal kin.

To contextualize the origins of the unique human social organization, with its hierarchical structure of individuals nested in households, anthropologists require insights about the strategies that people use to navigate the conflicts of interest that ensue from the concomitant social structures [12]. The targeting problem and related issues are frequently acknowledged but rarely the subject of empirical research. It is increasingly evident, however, that humans exhibit an evolutionary history of co-residence with affines and other genetically unrelated individuals, which is further combined with flexible dispersal patterns, marital arrangements and post-marital residence rules [13]. Adaptations to this social structure seemingly facilitated the emergence of affiliations at broader scales composed of multiple, mutually cooperative communities [2].

This paper addresses the hierarchical structure of human communities by examining the social support networks of indigenous Nicaraguan horticulturalists. This research builds on an emerging anthropological literature that focuses on the social networks of individuals [8,14,15]. To examine the effects of household membership on individuals' networks, I employ a multilevel statistical model with a random effects structure that distinguishes between individual-level and household-level effects and the extent of reciprocity evident at both levels of the hierarchy. Theoretically, the analysis is motivated both by Hamilton's [16] kin selection theory and Heider's [17] structural balance theory, which proposes that micro-level psychological processes shape dyadic interactions in response to third-party affiliations and conflicts. Additionally, the paper addresses how offspring conceived via extra-marital infidelity can potentially disrupt inter-household kinship networks, generating conflicts of interest that offset high consanguineal relatedness between households.

## 2. Predictions

### 2.1. Balance theory

It is common for behavioural ecologists to think in terms of dyadic relationships between two individuals, a tendency that relates in part to the preponderance of research on contingent reciprocity [18,19]. However, proponents of social network analysis often emphasize the contextualizing effects that third-party relationships have on dyads [20,21]. From this perspective, it is critical to understand how members of the dyad are embedded in triads and the social structure more generally. In particular, this research draws upon Heider's [17] balance theory, which asserts that social actors are motivated toward cognitively consistent triadic relationships in order to minimize psychological dissonance.

**Figure 1.**
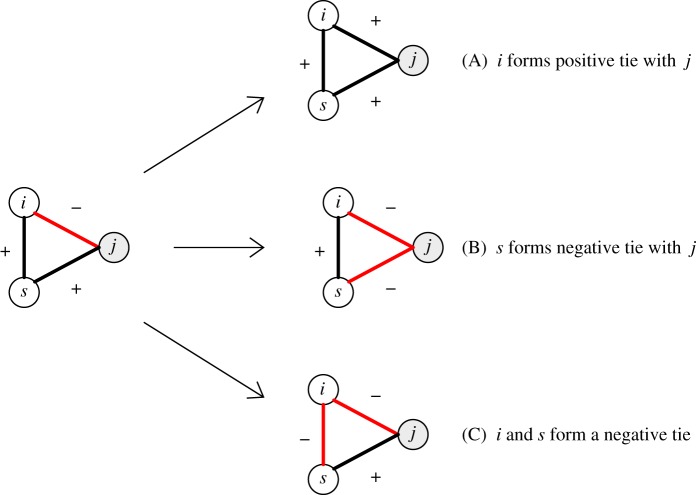
Depicted from the perspective of individual *i*, structural balance theory suggests that an imbalanced triad (left) can be resolved in three ways (right). The triad consists of three individuals, two of whom are from the same household (*i* and *s*) and a third from a separate household ( *j*). As described in the text, the prioritization of strong within-household relations is expected to make option C less likely than options A and B for resolving imbalances.

Balance theory can be readily conveyed via graphs. In figure 1, for example, consider an ‘imbalanced triad’ in which individual *i* maintains a negative tie to individual *j* and a positive tie to individual *s*, who resides in the same household with *i*. When *j* and *s* have a positive relationship, individual *i* experiences tension from this configuration. The discomfort can be resolved in multiple ways. Individual *i* could develop a positive tie with *j* or a negative tie with *s*, or perhaps coercively compel *s* to sever the positive tie to *j*. In all cases, the result is a ‘balanced’ triad. Colloquially, the logic of balance theory lends itself to sayings such as ‘A friend of a friend will be a friend’ and related variants [20].

When considering the relationships between members of different households, a key assertion of this paper is that within-household relations are an important arbiter of external ties. This is because individuals who share a household must coexist in close proximity, typically while negotiating the distribution of food and other resources and frequently working together toward collectively beneficial outcomes. Co-residents usually have privileged access to sensitive information about individuals and their resources, and considerable trust is needed to navigate these vulnerabilities. Therefore, maintaining strong, positive within-household ties will typically assume precedence over ties to members of other households. Extending the logic of balance theory, members of a household will consequently converge on a similar pattern of relationships to members of other households. As depicted in the graphs of figure 1, this suggests that household stability requires individuals *i* and *s* to maintain a positive tie, and, therefore, they will have symmetrically balanced ties to individual *j* from another household. Framed as a prediction, this in turn implies:

**Prediction 1.** If individual *i* from household *k* maintains a positive connection to individual *j* from household *l*, then other members of *k* are more likely to maintain positive connections to *j* and other members of household *l*. The same logic applies to the absence of ties, and statistically, this will result in a pattern of household-level correlations between *k* and *l*.

### 2.2. Kin selection theory

In small-scale societies, biological kinship is frequently regarded as the primary basis for social organization, and communities are, therefore, composed of networks of related individuals [2]. Ethnographic data frequently show that exchanges of resources are particularly common among kin, but as noted by Allen-Arave *et al*. [11], biases in altruism toward kin do not necessarily provide evidence for kin selection. That is, kin selection models consider not only the relatedness between individuals, but also the respective neediness of donors and recipients. Kin-directed altruism is evolutionarily adaptive when the costs to the donors are outweighed by the benefits to the recipients, discounted by their coefficient of relatedness. This model of kin selection is encapsulated via Hamilton's rule:

$$\sum_{j\in J}r_{j}b_{j}-c>0,$$

where *J* is the set of alters whose evolutionary fitness is affected by the behaviour of the actor, *r_j_* is the coefficient of relatedness between individual *j* and the actor relative to the population average, *b_j_* is the fitness benefit (or cost) to the fitness of individual *j*, and *c* is the effect of the behaviour on the actor's fitness. By considering the full range of alters affected by the behaviour, this notation is modestly more elaborate than the pairwise version of Hamilton's rule that is commonly employed by behavioural ecologists [22]. Extending the logic of Hamilton's rule to multiple alters is conceptually straightforward, however, as in J. B. S. Haldane's famous quip that he would sacrifice his life for two brothers or eight cousins, but not less [23]. Despite its overall familiarity to behavioural ecologists, kin selection theory is frequently characterized by conceptual misunderstandings [24]. For instance, it is sometimes overlooked that all parameters in Hamilton's rule can be positive or negative, including relatedness, which is defined relative to population averages, not simply pedigrees. In the case of human societies, however, pedigrees may provide a sufficient approximation of relatedness given the population mixing that typically results from sex-biased dispersal and other mechanisms that prevent high inbreeding [25].

Kin selection theory substantiates additional predictions about the multilevel interactions of individuals and households. Among individual-level dyads, pairwise altruism is conceptually straightforward, with assistance predictably flowing to needy individuals at marginal cost from close consanguineal kin. As noted in the discussion of balance theory, however, there can be conflicts of interest in the apportionment of help when multiple individuals and households are involved. Often, the respective conflicts and convergences of interests are predictable in light of the dyadic relatedness between individuals [26]. In general, greater average relatedness among group members tends to promote altruistic cooperation [27]. In terms of relationships among households, it is expected that higher relatedness between members of the respective households will promote greater inter-group altruism.

In multilevel human communities, kin selection theory merits empirical tests among both individual-level and household-level dyads, partly because targeting problems can sometimes be circumvented. As an alternative to sending packages of food to another household, for example, it may be possible to invite a subset of that household's members to visit and share in meals, effectively precluding consumption by unintended targets. It is possible that kin-directed altruism will be evident among both individual and households:

**Prediction 2a.** Among individual dyads, assistance will flow according to variation in need among closely related individuals.

**Prediction 2b.** Among households, members of relatively needy groups will receive assistance from individuals in prosperous groups, as biased toward household-level dyads exhibiting high average relatedness among its members.

### 2.3. Infidelity

Stringent interpretations of kin selection theory suggest that only the parameters of Hamilton's rule should influence altruistic behaviour toward kin. For instance, only the genetic relatedness between individuals is relevant, not categorizations related to social kinship. Thus, consanguineal kin should demonstrate equal concern for children regardless of the marital status of the children's parents. From this perspective, children born via adulterous relationships are not categorically different, and implicitly this perspective has led human behavioural ecologists to ignore such distinctions when calculating measures of inter-household relatedness.

There are reasons to expect that adulterous reproduction will result in behaviours that deviate from conventional predictions of kin selection theory. First, there is the problem of kin recognition. The consensus view is that humans and other primates rely primarily on associational cues to identify their genetic kin [28]. By treating one's mother as a point of reference, one can infer that individuals receiving maternal care from the mother are either full siblings or maternal half-siblings. Among most primates, it is unclear to what extent paternal half-siblings can distinguish their relatedness, though there is some evidence for recognition via phenotypic matching [29]. In human societies, it may be assumed that in most cases of adulterous reproduction, the children initially reside in their mother's household. In turn, this residence pattern would reduce the association and familiarity among the children and their paternal kin, potentially undermining the recognition mechanisms that facilitate kin-directed altruism. Although inferences about paternity and relatedness can be conveyed to individuals via language [30], it is not clear that learning about kinship ties via symbolic communication can rival association-based cues in terms of motivating kin-directed behaviour.

**Figure 2.**
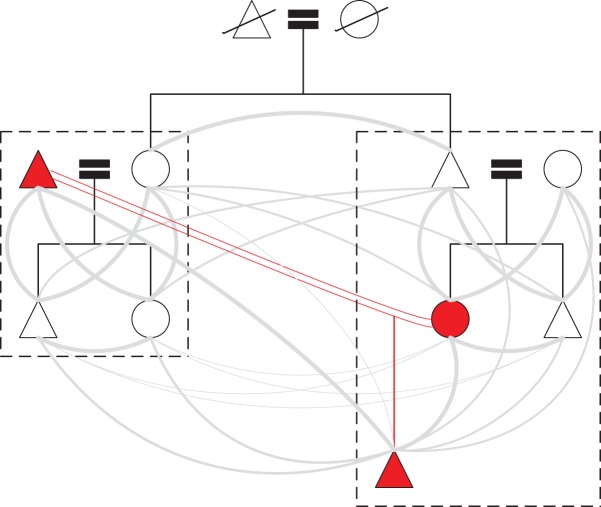
Consequences of infidelity on the relatedness of members of two hypothetical households. The image depicts an adulterous union between a woman and her aunt's husband, which results in a child (all three of those individuals are depicted in red). The dashed rectangles indicate household membership, and the grey arcs are sized proportionally to the degree of relatedness between the individuals. Note that, compared to other household members, the cuckquean has the lowest genetic relatedness to the child conceived during the adulterous relationship.

Adulterous reproduction also generates conflicts of interest that discourage inter-household cooperation.^1^ Cuckqueans are females whose mates have sired offspring with other females, and these women have little incentive to invest in their husbands' illegitimate offspring [31]. Yet, cuckqueans may often co-reside with individuals who exhibit relatively high relatedness to the illegitimate offspring (figure 2). Combining evolutionary reasoning with insights from balance theory, a straightforward prediction is that the cuckquean's negative inclination toward the illegitimate offspring will compel her to discourage investment in the child by other members of her household. Similarly, from the cuckquean's perspective, the mother of the illegitimate child may also be perceived as a continuing threat to solicit mating effort from the husband. As in the earlier prediction, within-household relationships are expected to trump inter-household relationships, and because of the cuckquean's negative inclinations toward these individuals, other members of her household are also expected to minimize relations with them and vice versa:

**Prediction 3.** When close consanguineal kin in separate households are related primarily because of adulterous reproduction instead of sanctioned unions, there will be declines in the assistance exchanged between members of these respective households.

## 3. Study site

This research took place in Arang Dak, a community of 279 indigenous Mayangna and Miskito horticulturalists in Nicaragua's Bosawas Biosphere Reserve. Regarding subsistence, swidden horticulture provides staple crops such as rice, beans, bananas and manioc. Hunting and fishing are important sources of dietary protein [32]. Residents also maintain livestock, including fowl, pigs, and cows, although cattle serve primarily as a store of wealth that can be liquidated on short notice. Inter-household food sharing is relatively common, albeit limited primarily to meat, not horticultural products [7]. Other common types of material assistance include labour exchanges, loans of dugout canoes and other technologies, and money lending in small amounts. For monetary income, many households rely on gold panning, an activity pursued by both men and women, often in small cooperative groupings. Additional sources of income include a limited number of teaching positions in the community school, which receives funding from the government. Also, a small number of individuals periodically obtain contracted work with external organizations. Overall, wealth inequality in the community is relatively high, as indicated by a Gini coefficient of 0.55 for household wealth.

The social organization is oriented around nuclear family households that often include other adult residents beyond the male and female household heads. For instance, it is common for young married couples to reside with the bride's parents for several years, generally until the couple has a second or third child [33]. Unmarried individuals are also common, including widows and widowers, bachelors, or divorcées, who collectively comprise about 30% of the population of residents over the age of 18. Depending on their generational status, these unmarried people typically reside with either their parents or adult children. There are no formal residence rules, but an uxorilocal bias is evident [34]. Descent is traced bilaterally, and terms derived from the prevailing Nicaraguan kinship system (i.e. the Eskimo system) have largely supplanted the indigenous kinship system [35]. Marriages between cousins are rare and generally discouraged. Fertility is high, with a total fertility rate exceeding seven births per woman [33].

The Mayangna and Miskito are relatively tolerant of informal and short-term sexual relationships among unmarried individuals. By contrast, marital infidelity potentially entails a social cost, which is particularly pronounced for women and comparatively modest for men. Replicating methods used by Scelza [36], a strong majority of male and female respondents report that sexual infidelity evokes greater jealousy than emotional infidelity (J. M. Koster 2017, unpublished data). In the absence of systematic methods [37], estimating the prevalence of extra-marital affairs is difficult because couples are secretive, but informal ethnographic observations suggest that extra-marital sexual relationships occur intermittently. Individuals seem to vary considerably in their propensity for engaging in extra-marital relations. When children are conceived from such unions, their status is not highly stigmatized, particularly in circumstances when philandering men couple with unmarried women. The genealogical relationships are openly acknowledged in most cases, and the children are assigned the father's surname.

## 4. Material and methods

In April 2013, all residents of Arang Dak who were at least 18 years old were invited to participate in the research (*n* = 108). As part of a broader interview on social support, participants were asked a question that focused on material support provided to the participants by others in the community. Specifically, participants were asked, ‘Who provides tangible support to you at least once per month?’ Relevant domains of support were then listed as examples and included sharing of food or firewood, lending of valuable items such as dugout canoes, and help such as uncompensated assistance clearing agricultural fields. This question combines the binary focus of Nolin's [38] measure of food sharing with the broader domains of support addressed by Kasper & Borgerhoff Mulder [8]. In the month before the interviews took place, the question was piloted with approximately 10 participants to finalize the phrasing in the indigenous languages. Network interviews were conducted using the ‘roster’ method [39]. That is, names of all potential alters (*n* = 107) were read aloud in random order to the participants, who verbally responded affirmatively when an alter met the criteria of the question.^2^

### 4.1. Analysis

For this analysis, only the relations between members of different households are considered, largely because intra-household relations exhibit minimal variation (over 90% of intra-household alters are nominated as providers of assistance). The modelling approach draws on and extends the multilevel Social Relations Model [40]. In addition to the model's conventional parameters, however, this extension includes random effects and correlations that reflect the hierarchical structure of individuals nested in groups, specifically their households.

Because the response variable is dichotomous, a probit link function is used to estimate the model, which is formulated as a latent-response model. Thus, underlying the observed binary response, *y_ik_*_,*jl*_, denoting whether or not individual *i* in household *k* provides support to individual *j* in household *l*, it is assumed that there is an unobserved or latent continuous response $y_{ik,jl}^{*}$ representing the propensity to provide support. If this latent response is greater than or equal to zero, then the observed response is 1; otherwise, the observed response is 0:

$$y_{ik,jl}=\left\{\begin{array}{ll} 1 & y_{ik,jl}^{*}\geq 0\\ 0 & y_{ik,jl}^{*}<0. \end{array}\right.$$

A linear regression Social Relations Model is then specified for the latent response $y_{ik,jl}^{*}$

$$y_{ik,jl}^{*}={\text{x}}_{ik,jl}\beta +a_{2k}+a_{1ik}+b_{2l}+b_{1jl}+c_{ik,l}+d_{k,jl}+h_{k,l}+u_{\left|ik,jl\right|},$$

where ${\text{x}}_{ik,jl}$ denotes the vector of covariates and **β** the associated vector of regression coefficients. The effects of actor *i* in household *k* reflect both a household-level random effect, *a*_2*k*_, which measures the extent to which all members of household *k* deviate from the average supportiveness, and *a*_1*ik*_, which measures the supportiveness of individual *i* across dyads. There are corresponding household-level, *b*_2*l*_, and individual random effects, *b*_1*jl*_, for recipient *j* in household *l*.

The random effects for households and individuals are assumed bivariate normally distributed with zero means and variances of $\sigma_{a2}^{2}$, $\sigma_{a1}^{2}$, $\sigma_{b2}^{2}$ and $\sigma_{b1}^{2}$, respectively. The corresponding covariances for households and individuals are denoted as *σ_a_*_2*b*2_ and *σ_a_*_1*b*1_. The correlations between node-level effects in the Social Relations Model are conventionally referred to as the ‘generalized reciprocity coefficient’ [7].^3^ I adopt this nomenclature for the hierarchical extension of the model, and the correlation between a household's giving and receiving tendencies is accordingly dubbed the *group-level generalized reciprocity correlation*, *ρ_a_*_2*b*2_, while the corresponding correlation for individuals is the *actor-level generalized reciprocity correlation*, *ρ_a_*_1*b*1_.

a2kb2k∼N00,σa22 σa2b2σb22,ρa2b2=σa2b2σa22σb22anda1ikb1ik∼N00,σa12 σa1b1σb12,ρa1b1=σa1b1σa12σb12.

The model also includes cross-level random effects for the interactions between actors and groups (i.e. households). The effect, *c_ik_*_,*l*_, measures the extent to which individual *i* helps members of household *l* in general, as distinct from her dyadic interactions with specific members of household *l*. There is an inverse effect, *d_k_*_,*jl*_, for the extent to which members of household *k* provide support specifically to individual *j*. These actor-group effects are assumed bivariate normally distributed with zero means, variances $\sigma_{c}^{2}$ and $\sigma_{d}^{2}$, and covariance *σ_cd_*. The correlation between these effects, *ρ_cd_*, is then dubbed the *actor-group reciprocity correlation*.

cik,ldl,ik∼N00,σc2 σcdσd2, ρcd=σcdσc2σd2.

There are also random effects, *h_k_*_,*l*_, for the directed dyadic relations between members of household *k* and household *l*. In other words, this effect measures the change in probability of assistance when the help is directed from one specific household to another, as transacted via individual members of the respective households. These group-level dyadic effects are assumed bivariate normally distributed with zero means, variance $\sigma_{h}^{2}$, and covariance *σ_hh_*. The correlation between these effects, *ρ_hh_*, is the *group-level dyadic reciprocity*, reflecting the extent of reciprocity between the respective households.

hk,lhl,k∼N00,σh2 σhhσh2, ρhh=σhhσh2.

Although a positive correlation is anticipated in this study, the parametrization also allows for negative household-level correlations.

Finally, there is a random effect, *u*_|_*_ik_*_,*jl*|_, for the symmetric individual-level dyads, composed of individuals *i* and *j*. These effects are assumed to be normally distributed with variance, $\sigma_{u}^{2}$.

$$u_{\left|ik,jl\right|}∼N(0,\sigma_{u}^{2}).$$

An *individual-level dyadic reciprocity* can be calculated by dividing this variance by the sum of the variance and the lowest-level variance of the probit model, which is constrained to 1 in order to identify the model.

$$\rho_{uu}=\frac{\sigma_{u}^{2}}{\sigma_{u}^{2}+1}.$$

This formulation implicitly assumes that the dyadic reciprocity among individuals is positive because $\sigma_{u}^{2}\geq 0$ [40,41].

As a way to characterize the relative importance of the different random effects as sources of unexplained variation in the dataset, variance partition coefficients (VPCs) are calculated for each model by dividing the estimated variance in question by the total variance as follows:

$$\begin{array}{rl} p_{a2} & =\frac{\sigma_{a2}^{2}}{\sigma_{a2}^{2}+\sigma_{b2}^{2}+\sigma_{a1}^{2}+\sigma_{b1}^{2}+\sigma_{c}^{2}+\sigma_{d}^{2}+\sigma_{h}^{2}+\sigma_{u}^{2}+1},\\ p_{b2} & =\frac{\sigma_{b2}^{2}}{\sigma_{a2}^{2}+\sigma_{b2}^{2}+\sigma_{a1}^{2}+\sigma_{b1}^{2}+\sigma_{c}^{2}+\sigma_{d}^{2}+\sigma_{h}^{2}+\sigma_{u}^{2}+1},\\ p_{a1} & =\frac{\sigma_{a1}^{2}}{\sigma_{a2}^{2}+\sigma_{b2}^{2}+\sigma_{a1}^{2}+\sigma_{b1}^{2}+\sigma_{c}^{2}+\sigma_{d}^{2}+\sigma_{h}^{2}+\sigma_{u}^{2}+1},\\ p_{b1} & =\frac{\sigma_{b1}^{2}}{\sigma_{a2}^{2}+\sigma_{b2}^{2}+\sigma_{a1}^{2}+\sigma_{b1}^{2}+\sigma_{c}^{2}+\sigma_{d}^{2}+\sigma_{h}^{2}+\sigma_{u}^{2}+1},\\ p_{c} & =\frac{\sigma_{c}^{2}}{\sigma_{a2}^{2}+\sigma_{b2}^{2}+\sigma_{a1}^{2}+\sigma_{b1}^{2}+\sigma_{c}^{2}+\sigma_{d}^{2}+\sigma_{h}^{2}+\sigma_{u}^{2}+1},\\ p_{d} & =\frac{\sigma_{d}^{2}}{\sigma_{a2}^{2}+\sigma_{b2}^{2}+\sigma_{a1}^{2}+\sigma_{b1}^{2}+\sigma_{c}^{2}+\sigma_{d}^{2}+\sigma_{h}^{2}+\sigma_{u}^{2}+1},\\ p_{h} & =\frac{\sigma_{h}^{2}}{\sigma_{a2}^{2}+\sigma_{b2}^{2}+\sigma_{a1}^{2}+\sigma_{b1}^{2}+\sigma_{c}^{2}+\sigma_{d}^{2}+\sigma_{h}^{2}+\sigma_{u}^{2}+1}\\ \;\;\text{and}\;\;\;p_{u} & =\frac{\sigma_{u}^{2}+1}{\sigma_{a2}^{2}+\sigma_{b2}^{2}+\sigma_{a1}^{2}+\sigma_{b1}^{2}+\sigma_{c}^{2}+\sigma_{d}^{2}+\sigma_{h}^{2}+\sigma_{u}^{2}+1}. \end{array}$$

The only deviation from the overall pattern is for the calculation of the lowest-level dyadic variance (*p_u_*), for which the numerator is the sum of the symmetric dyadic variance and the directed dyadic variance, which is constrained to 1.

Because this model combines principles of the Social Relations Model with a focus on the inter-group relationships of actors nested in groups, I refer to it as the Group-Structured Social Relations Model (GSSRM).^4^ Although this version of the model address only inter-group relations, a fuller elaboration of the modelling approach will simultaneously model both inter-group and intra-group networks.

### 4.2. Donor-oriented models

In addition to the recipient-oriented social support question described above, informants were also asked the inverse question, specifically to nominate the alters to whom they provide tangible support at least once per month. Asking informants to report on dyadic relationships as both donors and recipients of help is common in studies of social support networks, and researchers often elect to symmetrize the potentially divergent reports, inferring the existence of a directed tie if mentioned by at least one member of the dyad [8,38]. Such symmetrizing reduces the interpretability of the model, however, because the source of the nomination is no longer evident. Therefore, instead of symmetrizing, the respective responses are instead analysed separately, and the corresponding donor-oriented GSSRMs are presented fully in the electronic supplementary material and referenced intermittently throughout this paper.

### 4.3. Predictor variables

The models include predictor variables that are used to test the aforementioned hypotheses (table 1 for descriptive statistics). Those predictors include the following.

**Table 1.** Variable names, descriptions and summary statistics. Variables denoted with an asterisk were *z*-score standardized prior to the analysis.

variable	description	mean	s.d.	min	max
individuals (*n* = 108)					
age*	individual's age in years	34.48	13.83	18	75
male	indicator variable for individual's biological sex, as recognized locally	0.50		0	1
body mass index (BMI)*	ratio of weight in kilograms to squared height in metres	23.91	2.59	15.89	32.2
skin colour*	melanin index	51.55	4.65	43.4	67
individual dyads (*n* = 5602)					
degree of relatedness	Wright's coefficient of relatedness, as derived from genealogies	0.05	0.10	0	0.50
affinal relatedness	highest dyadic coefficient of relatedness to third-party alter in triads that contain an affinal relationship, excluding dyads with high consanguineal relatedness (see text for details)	0.06	0.11	0	0.50
godparental relationship	indicator variable to denote that one member of the dyad serves as a godparent for the other or at least one of that individual's children	0.04		0	1
household (*n* = 32)					
wealth	value of key possessions in thousands of Nicaraguan cordobas, log transformed	−0.92	1.07	−3.02	1.66
household dyads (*n* = 496)					
distance	inter-household distance in metres, log transformed	5.78	1.22	2.86	8.39
average relatedness	log transformed average relatedness between members of households, subsequently centred at the sample mean (−3.86) prior to analysis	−3.74	1.15	−7.67	−1.29
infidelity ties	indicator variable denoting household dyads with half-siblings stemming from adulterous matings	0.01		0	1

#### 4.3.1. Household wealth

This variable serves as a measure of neediness, as wealth is a significant predictor of child growth and dietary quality in this setting [33]. Wealth is calculated from an inventory of the households' key possessions, such as livestock and tools, and the market value of these items. Because this variable exhibits positive skew, it is log transformed prior to the analysis.

#### 4.3.2. Dyadic relatedness

This variable is a measure of genetic relatedness between individuals *i* and *j*, as derived from genealogical interviews and consequently constrained to lie between 0 and 1. Although it would be preferable to use genetic markers to calculate relatedness [42], the use of genealogies is a useful proxy for relatedness in this study because individuals frequently disperse during early adulthood, resulting in a well-mixed population.

#### 4.3.3. Average inter-household relatedness

When examining inter-household resource flows, it is common for human behavioural ecologists to use the average relatedness between the respective household members as the measure of kinship [11,41,43]. The rationale is that all household members potentially contribute to the decision to help another household [11]. That approach is replicated here, and the *average inter-household relatedness* was calculated for all household-level dyads.^5^ Because of its positive skew, this variable was log-transformed.

#### 4.3.4. Interaction terms

To test the second set of predictions related to kin selection, I construct three-way interaction terms from the main effects for the wealth of households *k* and *l* and the measures of relatedness (separate models include dyadic relatedness and inter-household relatedness, respectively). The use of interaction terms departs from previous research in this literature, which is characterized by the use of differences in need [11]. When using differences, however, it is not possible to include the main effects of the wealth of both household *k* and household *l* without introducing perfect collinearity into the model [44]. This analysis, therefore, follows the use of interaction terms by Hoff [45], and model predictions are generated for multiple combinations of wealth and relatedness in order to interpret these effects.

#### 4.3.5. Infidelity ties

Children who are conceived during adulterous relationships are readily acknowledged during the genealogical interviews. A binary, symmetric variable, *infidelity ties*, is used to denote household-level dyads in which such children reside in household *k* and half-siblings of these children reside in household *l*.^6^

### 4.4. Additional covariates

In addition to the covariates of interest, the models include a number of other predictors, which are briefly described along with expectations for the effects of these variables.

#### 4.4.1. Distance

The locations of the houses were recorded with a GPS device, and the coordinates were used to calculate the geographical distances between the households. Following reports from Nolin [38] and Power [15], *distance* is predicted to show a negative effect on helping, reflecting greater assistance among closer neighbours.

#### 4.4.2. Affinal *r*

Although relationships via mates and spouses periodically attract attention from behavioural ecologists [46], few researchers have evidently attempted to characterize the strength of these relationships. The variable, *affinal r*, is a new construct that intrinsically involves a third party, individual *s*, who is married to individual *i*. The value of *affinal r*, which is a symmetric covariate, is then the dyadic relatedness (as defined above) between individual *s* and individual *j*. For example, the *affinal r* between a man and his wife's mother is 0.5, and the value for the man and his wife's grandmother is 0.25. Although it is possible for *i* and *j* to have multiple third-party affinal ties if they are both married (such that two separate individuals can serve as individual *s*), the variable presently considers only the third-party *s* that maximizes the values of *affinal r* between *i* and *j*.^7^ Furthermore, an affinal tie through a third party does not preclude the possibility of a consanguineal tie between *i* and *j*. Technically, given the above definition, a mother would have an affinal tie to her child via her marriage to the child's husband. Therefore, when there is also a consanguineal tie between the two members of the dyad, then *affinal r* is set to zero unless its value is at least twice as large as the value of *dyadic relatedness* between *i* and *j*. These are admittedly arbitrary decisions and thresholds, but ethnographically, the resulting variable aligns with locally salient perceptions of kinship, which prioritize affinal relationships in the absence of comparably strong consanguineal ties. Affinal relatedness is not assumed to be subject to kin selection, but rather it serves as a proxy for the strength of social kinship ties stemming from marriages.

#### 4.4.3. Godparental relationship

Having converted to Catholicism, the indigenous populations have adopted the custom of enlisting godparents for their children's baptisms. A type of fictive kin, godparents are subsequently expected to provide both advice and occasional material assistance to their godchildren. A binary, symmetric variable for *godparental relationship* is used to denote dyads in which *i* or *j* serves as a godparent for the other member of the dyad or at least one of that individual's children.

#### 4.4.4. Age

The age of interviewed participants was elicited via conventional demographic methods [33]. Evolutionary predictions about the effects of age are informed by considerations of age-related productivity and reproductive value [47,48]. In small-scale societies, middle-aged individuals often exhibit greater economic production with gradual declines into senescent ages. Meanwhile, younger adults have higher reproductive value and, therefore, greater possible inclusive fitness benefits from alloparental investment. These considerations lead to predictions that younger individuals will receive assistance from more alters and that middle-aged adults will provide assistance to more alters. Linear and quadratic functions of *age* are included to account for the expected curvilinearity in the effects of age for individuals *i* and *j*, respectively.

#### 4.4.5. Sex

The Mayangna and Miskito have a pronounced sexual division of labour, with men devoting significantly more time to agricultural labour, hunting and gold panning while women devote more time to domestic chores and childcare [49]. Such work is often conducted in tandem with same-sex partners, so in addition to the main effects of *sex* for individuals *i* and *j*, the models also include an interaction term of these effects. Females serve as the reference category for the main effects.

#### 4.4.6. Body mass index

In the month before the interviews were conducted, anthropometric measurements were taken of all residents. The heights and weights of participants were used to calculate their body mass index (BMI), which is the basis for homophily in some Western populations [50]. To test for homophily, the main effects of *BMI* for actors and partners were included along with an interaction term [45]. To the extent that *BMI* is a proxy for individuals' well-being, these variables could also be interpreted as a test of need-based flows of assistance to needy individuals from those who are in better condition.

#### 4.4.7. Skin colour

Skin colour, and race more generally, are also a basis for homophilous social networks in Western populations [51]. Conceptions of race in Nicaragua are beyond the scope of this paper [52], but partly owing to historical circumstances, the darker-skinned indigenous populations endure substantial prejudice from Nicaragua mestizos. Indigenous parents sometimes respond favourably when their offspring are born with pale skin, as though this bodes well for their future prospects. Nicknames based on skin colour are also common. In this study, *skin colour* was measured using a reflectance spectrophotometer on the participants’ foreheads because of the forehead's high visibility during social interactions. Measurements are reported as the *M* index, which exhibits higher values for darker skin [53]. The main effects of *skin colour* for egos and alters were included as control variables along with an interaction term to evaluate homophily.

### 4.5. Estimation and models

All models are estimated using Markov chain Monte Carlo methods implemented in the *rstan* package [54]. In addition to modelling the empirical data, the models were estimated using simulated data, and this simulation shows that the model can adequately recover the correlated random effects. Overall, the models exhibit generally good mixing, as assessed via conventional diagnostics.

The analysis reported here includes multiple models, beginning with two ‘empty’ models that include only random effects and the intercept. These models provide insights into the structure of the data, including correlations between the random effects. The first empty model includes only the random effects for individuals and individual-level dyads, corresponding to a conventional Social Relations Model (see electronic supplementary material, file 1, for notation). The second empty model includes the full array of random effects, as notated above. Comparing these models shows the extent to which behaviour among individuals and dyads is patterned by the group-level effects. Subsequent models include covariates to test predictions, and a final model reflects an exploratory analysis of the data.

For interview-based network studies, the data are inevitably subject to inaccuracies, whether from varying semantic interpretations of the network questions or inaccurate and biased recall by the informants [55,56]. The questions in this study were pilot tested before data collection, but different interpretations of the questions possibly result in informants settling on heterogeneous thresholds for distinguishing helpful alters. This variation potentially results in high variances in the random effects for the informants who generated the nominations ($\sigma_{b1}^{2}$ in the recipient-oriented models and $\sigma_{a1}^{2}$ in the donor-oriented models). To assess the extent to which this variation affects other parameters in the model, I simulated data that introduced additional random error into the informant-level effects. This exercise suggests that the generalized reciprocity correlations (*ρ_a_*_2*b*2_ and *ρ_a_*_1*b*1_) are notably altered by informants' inaccuracy but that the dyadic correlations (*ρ_hh_* and *ρ_uu_*) are largely recoverable despite the simulated inaccuracy.

These results further suggest that interest in covariates for individuals as donors and recipients of help are best directed to the set of models in which the individuals are not the informants. For instance, interest in donor-level variables (pertaining to individual *i*) is best directed to the recipient-oriented models that are featured in the main text. Conversely, interest in recipient-level variables (pertaining to individual *j*) is best directed to the donor-oriented models in the electronic supplementary material. This recommendation assumes that the informants’ inaccuracy is averaged over the potential alters.

The raw data and statistical code for simulations and model fitting are included as electronic supplementary material files for replicative purposes.

## 5. Results

The variances and correlated random effects for all models are shown in table 2. Model 0 corresponds to a conventional Social Relations Model with only individual-level random effects, and this empty model suggests high dyadic reciprocity (*ρ_uu_* = 0.60) and that much of the variation in the data reflects dyadic variables $\left(\sigma_{u}^{2}=1.52,\;\;VPC=0.56\right)$. This result is consistent with reports that show high dyadic reciprocity among individuals.

**Table 2.** Model variances (*σ*^2^), correlations (*ρ*) and variance partition coefficients (*p*). Reported quantities are the posterior means (standard deviations in parentheses).

	parameter	model 0	model 1	model 2	model 3	model 4	model 5	model 6
$\sigma_{a2}^{2}$	house *k* variance		0.30 (0.15)	0.51 (0.15)	0.58 (0.15)	0.55 (0.15)	0.57 (0.15)	0.62 (0.15)
$\sigma_{b2}^{2}$	house *l* variance		0.14 (0.15)	0.16 (0.15)	0.18 (0.15)	0.15 (0.15)	0.18 (0.15)	0.16 (0.15)
$\sigma_{a1}^{2}$	actor *i* variance	0.81 (0.15)	0.88 (0.15)	0.37 (0.15)	0.41 (0.15)	0.40 (0.15)	0.39 (0.15)	0.38 (0.15)
$\sigma_{b1}^{2}$	partner *j* variance	1.17 (0.15)	1.92 (0.15)	2.21 (0.15)	2.42 (0.15)	2.37 (0.15)	2.28 (0.15)	2.33 (0.15)
$\sigma_{c}^{2}$	actor-group variance		0.47 (0.15)	0.52 (0.15)	0.59 (0.15)	0.57 (0.15)	0.54 (0.15)	0.59 (0.15)
$\sigma_{d}^{2}$	partner-group variance		0.89 (0.15)	0.98 (0.15)	1.08 (0.15)	1.06 (0.15)	1.01 (0.15)	1.11 (0.15)
$\sigma_{h}^{2}$	group-level dyad variance		1.78 (0.15)	0.53 (0.15)	0.57 (0.15)	0.57 (0.15)	0.48 (0.15)	0.39 (0.15)
$\sigma_{u}^{2}$	dyadic variance |*ij*|	1.52 (0.16)	0.36 (0.15)	0.43 (0.19)	0.52 (0.23)	0.51 (0.21)	0.47 (0.20)	0.51 (0.21)
*ρ_a_*_2*b*2_	group-level generalized reciprocity		0.12 (0.31)	0.09 (0.31)	0.09 (0.30)	0.08 (0.30)	0.10 (0.30)	0.10 (0.31)
*ρ_a_*_1*b*1_	actor-level generalized reciprocity	0.24 (0.09)	0.18 (0.11)	0.19 (0.12)	0.20 (0.12)	0.18 (0.13)	0.19 (0.12)	0.17 (0.13)
*ρ_cd_*	actor-group reciprocity		0.58 (0.09)	0.48 (0.10)	0.44 (0.10)	0.45 (0.09)	0.46 (0.10)	0.47 (0.09)
*ρ_hh_*	group-level dyadic reciprocity		0.88 (0.04)	0.61 (0.13)	0.63 (0.13)	0.63 (0.13)	0.58 (0.14)	0.49 (0.18)
*ρ_uu_*	actor-level dyadic reciprocity	0.60 (0.03)	0.26 (0.08)	0.29 (0.09)	0.33 (0.10)	0.33 (0.09)	0.31 (0.09)	0.32 (0.09)
*p_a_*_2_	house *k* VPC		0.04 (0.02)	0.08 (0.03)	0.08 (0.03)	0.08 (0.03)	0.08 (0.03)	0.09 (0.03)
*p_b_*_2_	house *l* VPC		0.02 (0.02)	0.02 (0.03)	0.02 (0.03)	0.02 (0.02)	0.02 (0.03)	0.02 (0.03)
*p_a_*_1_	actor *i* VPC	0.18 (0.02)	0.11 (0.02)	0.06 (0.01)	0.06 (0.01)	0.06 (0.01)	0.06 (0.01)	0.05 (0.01)
*p_b_*_1_	partner *j* VPC	0.26 (0.03)	0.25 (0.03)	0.33 (0.04)	0.33 (0.04)	0.33 (0.04)	0.33 (0.04)	0.33 (0.04)
*p_c_*	actor-group VPC		0.06 (0.01)	0.08 (0.01)	0.08 (0.01)	0.08 (0.01)	0.08 (0.01)	0.08 (0.01)
*p_d_*	partner-group VPC		0.12 (0.01)	0.15 (0.02)	0.15 (0.02)	0.15 (0.02)	0.15 (0.02)	0.16 (0.02)
*p_h_*	group-level dyad VPC		0.23 (0.02)	0.08 (0.02)	0.08 (0.02)	0.08 (0.02)	0.07 (0.02)	0.05 (0.02)
*p_u_*	dyadic VPC	0.56 (0.03)	0.18 (0.02)	0.21 (0.02)	0.21 (0.02)	0.21 (0.02)	0.21 (0.02)	0.21 (0.02)

That interpretation changes substantially with the introduction of the additional group-related random effects from the GSSRM. In this second empty model, Model 1, the group-level dyadic variance is high $\left(\sigma_{h}^{2}=1.78,\;VPC=0.23\right)$, and the *group-level dyadic reciprocity* is very high (*ρ_hh_* = 0.88). By contrast, *individual-level dyadic reciprocity* in this model declines considerably (*ρ_uu_* = 0.26). These results suggest that individuals cooperate in dyads largely because they belong to households that cooperate with each other. In other words, upon learning that someone from household *k* is helpful toward a member from household *l*, the model asserts a high probability that other members of those respective households are helpful toward each other. Because the correlation is positive, these flows of assistance are bidirectional, not unidirectional. This positive correlation remains strong as covariates in subsequent models explain the group-level dyadic variance, which suggests that this reciprocity is not necessarily attributable to predictors such as kinship and spatial propinquity. Overall, these results support the first prediction, as derived from balance theory.

A broader comparison of the VPCs suggests that the greatest source of variance relates to the nominations by individual *j* $\left(\sigma_{b1}^{2}=1.92;VPC=0.25\right)$, which stems in part from the sources of informant inaccuracy described above. The remaining variances and VPCs show that the random effects account for minor to moderate amounts of the variation. There is little variance for households as either providers $(\sigma_{k}^{2}=0.30,\;VPC=0.04)\;$ or recipients $\left(\sigma_{l}^{2}=0.14,\;VPC=0.02\right)$, suggesting that there are not households that consistently distribute or attract disproportionate amounts of assistance. The actor to group $(\sigma_{c}^{2}=0.47,\;VPC=0.06)\;$ and group to actor $\left(\sigma_{d}^{2}=0.89,\;VPC=0.12\right)$ effects are moderately variable, and these effects are correlated (*ρ_cd_* = 0.58). This positive *actor–group reciprocity* suggests that sometimes individuals share unique cooperative relationships with other households, independent of other residents from the individual's household. Finally, there is evidence for moderate heterogeneity in the number of nominations that individuals receive as providers of aid $\left(\sigma_{i}^{2}=0.88,\;\;VPC=0.11\right)$.

Subsequent models include covariates (table 3). Model 2 is a base model against which the kin selection models can be compared. Among other noteworthy results, this model shows that genetic kinship at both the individual level (*β*_18_ = 7.00) and household level (*β*_20_ = 0.65) predict helping.

**Table 3.** Model estimates for fixed effect parameters. Reported quantities are the posterior means (standard deviations in parentheses). Interaction terms are denoted by an asterisk.

	parameter	model 0	model 1	model 2	model 3	model 4	model 5	model 6
*β*_0_	intercept	−1.74 (0.16)	−2.07 (0.28)	−0.88 (0.71)	−0.79 (0.72)	−0.93 (0.73)	−0.90 (0.67)	−0.76 (0.69)
*β*_1_	male (actor *i*)			−0.43 (0.16)	−0.45 (0.17)	−0.44 (0.17)	−0.43 (0.16)	−0.49 (0.16)
*β*_2_	male (partner *j*)			−0.57 (0.32)	−0.58 (0.35)	−0.59 (0.34)	−0.58 (0.35)	−0.68 (0.33)
*β*_3_	male * male (actor–partner)			0.54 (0.11)	0.56 (0.12)	0.55 (0.12)	0.55 (0.12)	0.57 (0.12)
*β*_4_	age (actor *i*)			0.80 (0.12)	0.83 (0.13)	0.83 (0.12)	0.81 (0.12)	0.85 (0.12)
*β*_5_	age^2^ (actor *i*)			−0.23 (0.07)	−0.23 (0.07)	−0.24 (0.07)	−0.23 (0.07)	−0.22 (0.07)
*β*_6_	age (partner *j*)			0.06 (0.22)	0.06 (0.23)	0.05 (0.22)	0.06 (0.22)	0.09 (0.22)
*β*_7_	age^2^ (partner *j*)			0.09 (0.14)	0.09 (0.15)	0.09 (0.14)	0.09 (0.14)	0.09 (0.15)
*β*_8_	BMI (actor *i*)			0.03 (0.09)	0.02 (0.09)	0.02 (0.09)	0.02 (0.09)	0.03 (0.09)
*β*_9_	BMI (partner *j*)			−0.15 (0.18)	−0.16 (0.19)	−0.16 (0.18)	−0.15 (0.18)	−0.18 (0.19)
*β*_10_	BMI * BMI (actor–partner)			−0.01 (0.03)	−0.02 (0.03)	−0.01 (0.03)	−0.01 (0.03)	−0.01 (0.03)
*β*_11_	skin colour (actor *i*)			−0.08 (0.09)	−0.08 (0.09)	−0.08 (0.09)	−0.08 (0.09)	−0.09 (0.09)
*β*_12_	skin colour (partner *j*)			−0.11 (0.17)	−0.12 (0.18)	−0.11 (0.18)	−0.12 (0.17)	−0.13 (0.17)
*β*_13_	skin colour * skin colour (actor–partner)			−0.01 (0.04)	−0.01 (0.04)	−0.01 (0.04)	−0.02 (0.04)	−0.02 (0.04)
*β*_14_	godparental relationship			1.01 (0.18)	1.06 (0.19)	1.05 (0.19)	1.02 (0.18)	1.06 (0.19)
*β*_15_	wealth (actor house *k*)			0.02 (0.15)	0.12 (0.17)	0.00 (0.16)	0.02 (0.15)	0.04 (0.16)
*β*_16_	wealth (partner house *l*)			0.00 (0.19)	0.12 (0.20)	0.01 (0.21)	0.01 (0.19)	0.02 (0.19)
*β*_17_	distance			−0.23 (0.10)	−0.24 (0.10)	−0.24 (0.10)	−0.23 (0.09)	−0.22 (0.09)
*β*_18_	degree of relatedness			7.00 (0.78)	5.92 (0.91)	7.23 (0.80)	7.21 (0.79)	2.38 (1.24)
*β*_19_	affinal relatedness			3.16 (0.47)	3.28 (0.50)	3.27 (0.48)	3.27 (0.47)	0.43 (0.95)
*β*_20_	average relatedness			0.65 (0.09)	0.67 (0.10)	0.78 (0.15)	0.69 (0.10)	0.60 (0.10)
*β*_21_	wealth * wealth (house *k* and house *l*)				0.08 (0.06)	0.01 (0.06)		
*β*_22_	degree of relatedness * wealth (house *k*)				−0.89 (0.48)			
*β*_23_	degree of relatedness * wealth (house *l*)				−1.13 (0.49)			
*β*_24_	degree of relatedness * wealth * wealth				−0.45 (0.38)			
*β*_25_	average relatedness * wealth (house *k*)					0.13 (0.08)		
*β*_26_	average relatedness * wealth (house *l*)					0.05 (0.08)		
*β*_27_	average relatedness * wealth * wealth					0.06 (0.06)		
*β*_28_	infidelity ties						−2.13 (0.53)	−2.14 (0.53)
*β*_29_	degree of relatedness * average relatedness							2.94 (0.67)
*β*_30_	affinal relatedness * average relatedness							1.77 (0.55)

**Figure 3.**
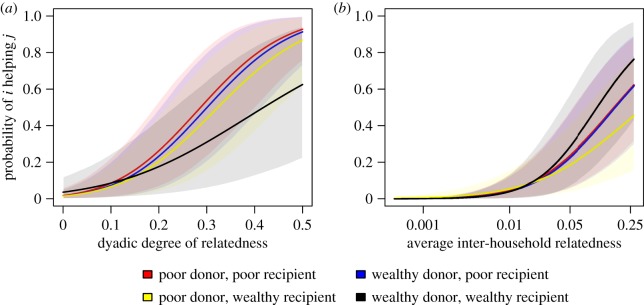
Predictions of Model 3 (*a*) and Model 4 (*b*) showing interactions between kinship and the household wealth of donors and recipients of aid. For high and low wealth, the predictions are based on values of wealth at the 90th and 10th percentiles from the empirical distribution, respectively. Other predictors are held constant at their means or reference values. Shaded areas depict the 89th percentile confidence intervals around the model predictions.

Models 3 and 4 provide tests of kin selection among individual-level dyads and household-level dyads, respectively. Figure 3 plots the predictions of these models, neither of which aligns clearly with predictions of kin selection theory. That is, the models do not show that individuals in wealthy households are especially likely to assist closely related individuals or groups of kin in poorer households. In other words, the main effects of kinship continue to be strong, but there is little evidence for moderating effects related to differences in wealth.

To test prediction 3, Model 5 includes the binary covariate, *infidelity ties*. In support of the prediction, this variable exhibits a strong negative effect on helping (*β*_28_ = −2.13). Holding all other covariates at their means or reference values while supplying a value of 0.25 for Wright's *r* (equivalent to a woman and her maternal aunt) the model predicts a reduced probability of helping from 36% to 2%.

### 5.1. Other covariates

*Distance* consistently exhibits a negative effect on helping, as closer neighbours reportedly provide more assistance (electronic supplementary material, figure 1). The variable for *godparental relations* also exhibits the predicted effect, and when holding all other covariates constant at their means or reference levels, dyads with a godparental tie show an 18% increase in the probability of a nomination. Regarding the *sex* of the individuals, women nominate more providers of assistance, and they are particularly likely to nominate other women as sources of aid (electronic supplementary material, figure 2). The *age* of individual *i* is predictive of helping, with the model primarily suggesting that people younger than about 30 years older are less helpful than their older counterparts (electronic supplementary material, figure 3). By contrast, the *age* of individual *j* exhibits little effect on helping nominations, possibly stemming from the informant inaccuracy mentioned above (and hence readers are directed to the donor-oriented models below for insight into age-related effects of individual *j*). Finally, the variables pertaining to the individuals' morphological phenotypes, whether *BMI* or *skin colour*, seemingly have little effect on the helping relations of individuals in this community.

### 5.2. Exploratory model

An additional model, Model 6, is reported alongside the previously described models. In the interest of reproducible science [57], this is disclosed as an exploratory model, not one that was planned prior to beginning the data organization and analysis. It was motivated by the results of Model 4, which shows strong effects of relatedness both among individuals (*degree of relatedness* and *affinal r*) and households (*average inter-household relatedness*). The model includes cross-level interactions between the respective main effects.

**Figure 4.**
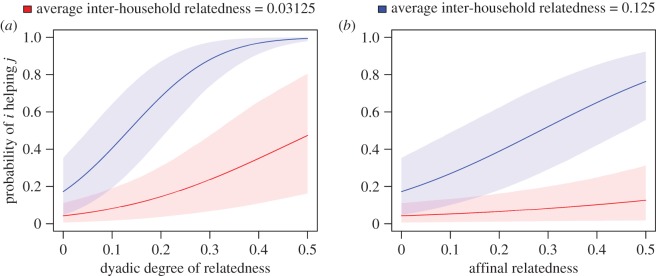
Predictions of Model 6 showing the interactions between average inter-household relatedness and dyadic relatedness (*a*) and affinal relatedness (*b*). Model predictions for low and high inter-household relatedness are based on values of 0.03125 and 0.25, respectively. Other predictors are held constant at their means or reference values. Shaded areas depict the 89th percentile confidence intervals around the model predictions.

In this model, household-level relatedness exhibits a positive moderating effect on both *degree of relatedness* and *affinal r* (figure 4). In other words, when members of their respective households exhibit high genetic relatedness, then individuals with strong consanguineal or affinal kinship ties have a higher probability of reporting assistance. As a practical example, consider two full siblings who move to the community and separately marry and cohabitate with partners who reside in households with low average relatedness. In these circumstances, the model predicts that these siblings and their spouses would help each other less than if the couples were to form new households composed primarily of members who exhibit stronger kinship ties (siblings, nieces, nephews, cousins, etc.).

### 5.3. Donor-oriented models

The models for the inverse question, in which informants nominate alters whom they have helped, are reported in electronic supplementary material, tables S1 and S2. The general concordance of the main models with their corresponding supplemental models suggests that the primary results of this study are robust to the directed framing of the network question. In particular, these models show high values for group-level dyadic reciprocity and variance, a bias to helping kin albeit with little effect of relative wealth, and a decline in helping among households with *infidelity ties*. Differences between the models are evident primarily for the effects of age. In the donor-oriented models, there is an increasing effect of donor's age across the lifespan, as younger adults report that they assist fewer alters than their older peers. Age is also a positive predictor of receiving help, as older individuals receive more nominations as recipients of aid than younger adults.^8^

## 6. Discussion

### 6.1. Structural balance and kinship

This study shows that dyadic cooperation among individuals is structured according to the households in which they reside. As seen in the high variance and *group-level dyadic reciprocity correlation*, individuals who assist each other frequently reside in households that exhibit broad tendencies to cooperate and reciprocate assistance. These results are consistent with structural balance theory, particularly if co-residence in households serves as a ‘tie-breaker’ of sorts, leading individuals to prioritize stronger relationships with fellow household members over network connections to other people. Given the trust and coordination that may be required to share domiciles, this prioritization of housemates will plausibly be a common outcome in many human communities. This is not to presume the absence of intra-household conflict. However, prolonged and intense conflict among adult housemates may be rare given the potential and motivation for at least one of the individuals to vacate the household and reside elsewhere. This potential for fissioning will further ensure that at any given time in most households, co-residents will be exhibiting positive dyadic relationships. In turn, housemates are expected to maintain a structurally balanced portfolio of network connections to members of other households. These dynamics seem to explain the prominence of household-level dyadic relationships among the Mayangna and Miskito.

These results do not presuppose the unimportance of an individual-level perspective on social support networks. Even after accounting for household-level random effects and covariates, the *individual-level dyadic reciprocity* correlation remains moderately strong. This result is consistent with other recent studies that have shown positive dyadic reciprocity among individuals [8,15]. Also not to be overlooked is the *actor–group reciprocity* correlation, which remains relatively strong in all models. This suggests that in some cases there are unique relationships between individual *i* and the members of other households (household *l*), as unique from the co-residents in individual *i*'s household (household *k*). The variances of the corresponding random effects are not as high as others in the model, suggesting that the importance of these actor–group relationships is less prominent than other sources of variation. Yet, the persistence of the correlation across models suggests that the covariates in the models, including variables for kinship, explain relatively little of this variation. Additional theorizing is therefore needed to conceptualize circumstances in which individuals would deviate from housemates in terms of relationships with members of other households. These deviations pertain both to scenarios in which individual *i* is unique in maintaining positive relationships with another household and also to scenarios in which the individual is unique for *not* joining housemates in sharing positive ties with another household. For the network data in this Nicaraguan community, an informal retrospective review of the respective varying intercepts suggests few commonalities among the actor–group combinations that exhibit noteworthy values for either of those two scenarios.

In small-scale societies, kinship is a primary determinant of social structure [59], and this analysis further confirms the importance of kinship, whether consanguineal, affinal or fictive. In this setting, the importance of kinship as a predictor of helping is further magnified by the evident unimportance of phenotypic variables that predict homophilous social networks in industrialized settings, such as similarity in skin colour and body mass. On the other hand, whereas kinship is an unambiguous predictor of helping in this study community, there is little evidence that differences in need moderate the effects of kinship. In other words, the results do not reveal flows of assistance from wealthier individuals to comparatively poorer kin. This analysis therefore introduces additional uncertainty into the literature on kin selection as the basis for resource transfers in small-scale societies [60]. That is, a number of studies have shown a predictive effect of genetic kinship [61,62], and null findings for the importance of kinship tend to be characterized by small sample sizes [41,63] or to focus on demographically atypical subsets of the population [64,65]. However, as noted by Allen-Arave *et al*. [11], a bias to helping kin provides unambiguous support for kin selection theory only when genetic relatedness interacts with differences in need. To date, few studies of inter-household cooperation have included the interaction terms needed to test for this effect, and empirical support for kin-biased altruism is limited primarily to a recent study among the Tsimane of Bolivia [43, cf. 11].^9^

What explains the lack of clear support for predictions of kin selection theory in this analysis? One possibility is that variation in household wealth is an unrepresentative indicator of need in this setting. Household wealth has figured prominently in recent studies and debates [8,67], and as noted, wealth is predictive of nutritional indicators among the Mayangna and Miskito. However, because of illnesses or unexpected subsistence failures, need can vary over brief timeframes in ways that are only partially mitigated by maintaining stores of wealth [68]. The month-long timeframe evoked by the network questions in this study may have been too coarse to capture the short-term fluctuations in need that would motivate unidirectional kin-directed altruism.

Another possible explanation is that among the Mayangna and Miskito, the existence of a household implies that it can subsist as a largely self-sufficient residential unit. In other words, it is uncommon to observe households that require substantial subsidies from other households. Although previous research suggests that large harvests of fish and game are routinely shared between households [7], food diaries indicate that in the average household, less than 10% of the caloric intake stems from inter-household gifts (J. M. Koster 2017, unpublished data). This figure contrasts with societies such as trekking Ache foragers and Hiwi foragers, for whom substantially more calories originate from individuals outside nuclear families [64,69]. Among the Mayangna and Miskito, young couples sometimes construct and briefly occupy a new home before subsequently abandoning the dwelling and re-establishing residence with older relatives, typically the woman's parents. As a rationale for these decisions, local informants propound that the young couples were unable to feed themselves. Such residence patterns ensure that virtually all households are able to meet their basic subsistence needs and that inter-household assistance among kin is primarily oriented toward mutualistic and reciprocal exchanges.

By contrast, there is considerable potential for need-based transfers among members of the same household. Needy individuals and families seek residence in households of kin that can support them, and married couples regularly open their homes to their older adult children, including divorcées and their offspring, and to elderly parents who can no longer maintain separate households. Economic labour is primarily a male activity in this setting, and time allocation observations suggest that reproductive-aged men assume responsibility for most of the productive labour that sustains the households and extended families [49,70]. Qualitatively, the resulting distribution of resources from adult Mayangna and Miskito men to needy co-resident kin is similar to resource flows that characterize men's labour and distributions in other small-scale societies [43,61]. Overall, as opposed to the comparatively robust literature on inter-household support networks, such considerations imply that behavioural ecologists should devote greater attention to intra-household transfers as the locus of kin-directed altruism.

This research suggests that, in addition to its central importance for the cooperative production and allocation of resources, membership in a household has consequences for individuals' social networks. Assuming that structural balance with fellow household members is a priority, individuals sometimes have to de-emphasize social relations that deviate from the macro-level pattern between the households. Choices about where to reside therefore have potentially dramatic effects on individuals’ fitness and related outcomes. Prevailing evolutionary perspectives suggest that households are inherently unstable, subject to change when individuals in the group can realize higher fitness by departing the group [71]. Yet, except for studies that address the consequences of divorce [72], human behavioural ecologists have devoted surprisingly little attention to the fluidity of household membership and dispersal decisions [73,74]. Among sociologists and demographers, analogous research on this topic is methodologically straightforward [75–77]. Anthropologists who collect longitudinal data on co-residence and dispersal could employ similar methods while incorporating predictor variables that test specific predictions from evolutionary theory, including models of competition and conflict among kin [78]. In this ethnographic setting, young Mayangna and Miskito adults who co-reside with parents initially seem to incur few reproductive costs, as virtually all couples have their first children while sharing a household with parents. On the other hand, in-law conflict is among the most oft-cited reasons for the dissolution of early marriages. The extent to which this and related social tradeoffs manifest in different settings can potentially explain cross-cultural variation in household composition, which merits greater research from anthropologists who are interested in kin selection theory.

To reiterate, assistance between members of different households is patterned by consanguineal kinship. A novel finding of this study, however, is that relatedness originating via infidelity is associated with a reduction in aid. A standard evolutionary explanation for this result would be that ancestral kin recognition mechanisms are ill-equipped for these circumstances. In other words, because children born out of wedlock typically reside with their mothers and separately from their fathers, paternal kin have fewer opportunities to interact with these children, thus impeding their recognition as kin. An alternative explanation focuses on the cuckquean's motivation to discourage investment in the offspring born via her husband's philandering [79]. That is, because she is not closely related to the resulting offspring, the cuckquean has incentives to dissuade her husband, offspring and other kin from investing in these children, preferably redirecting investment toward her genetic kin. She also has incentives to discourage additional mating effort by her husband toward the adulterous paramour. In keeping with structural balance theory, the cuckquean's negative attitude toward the paramour and her offspring would then result in similar negative relations by other members of her household. Ethnographically, both explanations receive support from observations and interviews at this field site. For instance, informants indicate that they harbour stronger familial ties toward individuals with whom they resided as children. At the same time, adulterous relations discernibly result in persistent enmity and avoidance behaviour among the persons involved. Additional research is needed to disentangle the relative importance of these factors as explanations for the reduction in assistance among households that are linked by adultery-related kinship ties.

Another contribution of this research is its secondary focus on social kinship, as seen in elevated probabilities of assistance among affinal kin and dyads that feature godparental connections. These variables were included primarily as control variables, but they exhibit strong and consistent effects that accentuate the multidimensionality of human kinship ties. As compared to genetic kinship, it is more difficult to infer causal relationships because of the endogeneity that characterizes these social networks [80]. For instance, do godparents provide help because of the role they fill, or were they selected as godparents primarily because of the assistance they were already predisposed to render? Resolving such questions may require a combination of longitudinal data, natural experiments and statistical methods that have previously been rare in anthropological research [81].

Meanwhile, the strong effects of *affinal relatedness* expose the lack of theoretical attention to cooperation among affinal kin, which has only intermittently piqued the interest of behavioural ecologists [63,82]. Much like perspectives on the overlapping reproductive interests of spouses [83], the incentives for helping other affinal kin may be regarded as self-evident, namely that it can be beneficial to assist alters who are predisposed to help one's genetic kin. Although affinal kin typically exhibit low genetic relatedness, they frequently have convergent interests in terms of their relatedness to third-party connections. For example, a woman might help her brother-in-law, knowing that he is predisposed to parlay that assistance into benefits for her consanguineal nieces and nephews. The importance of these overlapping interests may be particularly pronounced in human societies given the ubiquity of resource transfers, which afford abundant opportunities for secondary distributions [84]. If affinal kin can be trusted to bring resources back to their households, thus benefitting everyone who resides in the household, then in many scenarios it may be advantageous to rely on that pathway for assisting kin instead of attempting to direct resources to specific individuals. New theoretical approaches are needed to illuminate the circumstances in which altruism toward affines can be adaptive relative to alternative strategies.

Consideration of overlapping, third-party connections motivated this paper's exploratory model, which shows positive interaction effects between the relatedness of individuals and the average relatedness of their respective households. A possible interpretation of this result again relates to the household-level extension of structural balance theory, as individual dyads are more likely to maintain positive relationships when members of their respective households are likewise predisposed toward positive ties, in this case because of high relatedness. Given that many resources are consumed collectively within households, this result is generally consistent with kin selection theory, too. Holding other variables constant, if donors of resources were to choose between two possible recipients, then preferences should emerge for recipients who will subsequently share those resources with individuals who are highly related to the original donors. Interestingly, in addition to the positive interaction term for close consanguineal kin, the model shows that assistance rendered among close affinal kin is moderated by household-level relatedness. In the absence of high average relatedness among the respective household members, individuals are unlikely to provide assistance to close affinal kin who reside in other households. Such results suggest that cooperation among kin exhibits group-level dynamics that transcend the basic pairwise applications of Hamilton's rule by behavioural ecologists [85].

A general implication of this study is that concerns about the targeting problem have perhaps been overstated.^10^ As seen in the high estimates of household-level dyadic variance and reciprocity, there is considerable overlap in the helping relations among the individual members of respective households, and much of this variation is explained by kinship. For future research on inter-household cooperation, in which only household-level networks and predictors are available, this study, therefore, suggests that prevailing methods will often suffice when conflicts of interest among kin are similarly modest [12]. However, instead of relying on a single measure of household-level kinship, whether average relatedness or the closest genetic tie between the households, researchers should consider the broader dimensionality of kinship and potentially multiple measures of inter-household relatedness [38]. In some cases, it may be appropriate to include multiple terms for kinship in the same statistical model, particularly given the results presented here on the interaction between the dyadic relatedness of individuals and the relatedness of their respective households.

### 6.2. The analysis of social networks

A secondary goal of this paper is to propose and advance the GSSRM as a beneficial statistical method for social networks in which individual nodes are discretely nested in higher-level groups (e.g. households). Similar multilevel communities are evident among non-human primates, including the conglomerations among members of one-male groups found among geladas and several colobine species [86]. Among humans, such multilevel nesting is ubiquitous and in addition to household membership, examples include individuals who are members of discrete unilineal descent groups [26]. Examples of broader social scientific applications include the multilevel networks of teachers who work in different departments or schools [87] or corporate employees who are nested in distinct offices or divisions [88]. The GSSRM, which can equally be extended to count or continuous response variables, elucidates the statistical dependencies that ensue from this multilevel structure, providing a comparatively insightful perspective on the sources of variation in individuals' social networks.^11^

This study, therefore, challenges the assumption of dyadic independence that has frequently typified research by behavioural ecologists. That is, whereas prevailing methods typically account for the correlation in the directed relations of two nodes [7], those relations are otherwise assumed to be independent of their respective connections to other nodes in the network. By incorporating terms that reflect triadic dependencies, anthropologists using exponential random graph models (ERGM) have recently shifted the focus toward the broader social structure that impacts dyadic relationships [15,89]. This paper includes models of inter-household cooperation that advance this scholarship further by revealing clustering among household-level dyads, which is the outcome predicted by structural balance theory when the maintenance of positive ties to housemates is prioritized over alternatives. However, the GSSRM and ERGM are not the only statistical approaches that model the structural dependencies of network data. On the contrary, numerous modelling alternatives are available that explicitly model the latent social structures that frequently typify social networks [90,91]. In the hands of behavioural ecologists, the use of these methodological tools can potentially prompt new theorizing and empirical tests about social behaviour. There is a risk, however, that statistical models can be conflated with the mechanisms that generate the data, and refined observational methods are also necessary to substantiate the behaviours that purportedly effect a given social structure.

## 7. Conclusion

This study revisits longstanding questions of social theorists. It has been more than a century since Weber [92] questioned the extent to which individual behaviours are contextualized and determined by their embeddedness in social networks. Generations of social anthropologists have grappled with this question, ranging from the work of Evans-Pritchard [93] on triadic imbalance among Azande families to studies of the structure of kinship relations among G/wi bushmen [94]. In recent decades, however, research on kinship and social networks has been largely abandoned by most cultural anthropologists, for whom kinship studies are purportedly passé [95]. Human behavioural ecologists have maintained strong interest in kinship and social organization [13,34,96], but perhaps motivated by dyadic formulations of Hamilton's rule, much of the empirical research focuses on pairwise interactions and altruism, not the broader social structure that shapes dyadic relations. With the development of the GSSRM and related statistical approaches, models are possible that simultaneously examine dyadic behaviour and the network structures that contextualize the dyads.

This study shows high similitude among the social networks of household members, who tend to maintain cooperative relationships with the same sets of alters, who are themselves clustered in households. There is a temptation to generalize from this result to broader debates about prehistoric social organization and human evolution, arguing for the central importance of the household as a fundamental unit of social organization. For instance, centred around normatively monogamous married couples, Mayangna and Miskito households exemplify aspects of the strongly bonded multifamily groups described in Chapais's [97] treatise on the distinctive multilevel social structure of human communities. The anthropological legacy, however, attests to the extraordinary diversity of human social organization, and it is not clear that the strong household-level effects in this study would be as evident in other settings. Among other factors, the similitude of household-level social networks conceivably responds to societal variation in descent rules, the prevalence of polygamy, post-marital residence rules, and sources of conflict among wives, husbands and other household members. Robust cross-cultural generalizations about the hierarchical structures of human communities will become increasingly feasible as anthropologists adopt the methodological tools to disentangle the social networks of individuals and their households.

## Supplementary Material

Supplemental notation, figures, and tables

## Supplementary Material

Data

## Supplementary Material

Statistical code for analysis

## Supplementary Material

Simulation code

## Footnotes

1 This example assumes the perspective of the cuckquean, but the logic would equally hold for cuckolds, potentially with variation related to the residence patterns.

2 Although there were 108 adult residents in the community, only 106 were interviewed because two adults had absconded temporarily to another community to engage in an adulterous relationship (the man was married whereas the woman was not). The dataset thus includes the interviewees' reports on the helping provided by those missing two individuals, but not the reports from the missing individuals. In other words, there are reports about the help from 108 donors to 106 recipients.

3 In the parlance of social network analysis, the generalized reciprocity correlation implies a roughly comparable correlation between in-degree centrality and out-degree centrality.

4 Although rare among behavioural ecologists, it is common for statisticians who study social networks to assign names to their statistical approaches in order to facilitate scholarly discourse about the respective models.

5 Although they were not interviewed as part of the study, household members younger than 18 were included in the calculations of inter-household relatedness.

6 For example, imagine that a male from household *l* has an illegitimate child in household *k*, then the household-level dyad would be coded as ‘1’ for *infidelity ties*. Furthermore, imagine that the philandering man has an adult daughter living in household *m*. In that case, the |*k*,*m*| dyad is likewise coded as having *infidelity ties*.

7 In one case, for example, a man's wife's niece married the man's brother, a full sibling. When treating the man's wife as the affinal tie, the *affinal r* between the man and the niece is 0.25. By contrast, when treating the niece's marriage to the brother as the affinal tie, the value of *affinal r* is 0.50 because the man and the niece are siblings-in-law. The maximum value of 0.50 is the value used in the present analysis.

8 To some extent, this result might reflect an emic norm of respecting community elders. A recent isotopic analysis of hair samples, however, shows that older individuals at this study site consume a nutritionally enhanced diet relative to younger individuals [58]. Although alternative mechanisms have not been ruled out, this finding provides indirect evidence that norms to assist one's elders potentially result in increased altruistic behaviour toward older individuals.

9 In an earlier paper, I had used interaction terms in an analysis of inter-household meat sharing, and that analysis showed that shared meat flows from productive households to close kin in less productive households [66]. In a related paper [7], I did not use interaction terms when analysing a related subset of the data. While employing the social relations model (SRM) for count data [7], a recent reanalysis of the data with interaction terms that parallel those in this study reveals little evidence for kin-directed altruism. In other words, there is little statistical support for the interaction terms, and that applies whether using the main effects for the productivity of *i* and *j* (and then including three-way interactions with relatedness) or when calculating the imbalance of productivity, as in other analyses [43,66]. The ‘significant’ effects in the 2011 paper evidently occurred for two reasons. First, unlike the SRM for count data, the matrix permutation (MRQAP) models in the 2011 paper assume a continuous response variable and they did not adequately account for the statistical non-independence of nodes and dyads; relatedly, significant effects for the interaction terms are evident when specifying a continuous response with the random effects or when omitting the random effects from a conventional Poisson regression. Second, the earlier paper did not include the full set of covariates from the 2014 paper, such as measures of wealth and the ownership of pigs by the respective households. Without these covariates, a Poisson SRM shows stronger and significant effects for the interaction terms. The 2011 paper, therefore, appears to be a cautionary tale about interpreting underspecified models using outdated statistical methods. In retrospect, the reanalysis suggests a strong effect of kinship on sharing, but kinship is evidently not moderated by differences in need between households. The data for the reanalysis are available as a supplement to the 2014 paper.

10 At least to some extent, informants are cognizant of the targeting problem. Following the data collection, multiple Mayangna and Miskito participants spontaneously volunteered perspectives on their social networks with statements such as, ‘You know, when individual *i* comes back with hunted game, I know the only reason I receive a piece is because his wife is my aunt and she arranged for me to get a portion. But I went ahead and nominated individual *i* because he probably could have objected to the gift if he felt strongly about it’.

11 Other methodological advances remain pending. For instance, the current version of the GSSRM assumes positive dyadic reciprocity among individuals *i* and *j*, but the model could be reformulated to permit negative dyadic reciprocity. Also, there is substantive interest in the moderating effect of kinship on dyadic reciprocity, but the statistical methods to examine such effects remain undeveloped (P. Hoff 2017, personal communication). Also, in this study the donor-oriented data were analysed separately, but there is a need for statistical methods that incorporate all available data without relying on symmetrizing the data.

## Data accessibility

The data and coding script that support this article have been uploaded as part of the electronic supplementary material.
