# Supplementary material for: Family ties: the multilevel effects of households and kinship on the networks of individuals
Source: R Soc Open Sci. 2018 Apr 18;5(4):172159. doi: 10.1098/rsos.172159 (PMC5936935; doi:10.1098/rsos.172159)
Supplement: Supplemental notation, figures, and tables [file rsos172159supp1.pdf]

Supplemental File 1. Statistical notation for a conventional Social Relations Model for binary data, assuming positive dyadic reciprocity.

$$y_{i,j} = \begin{cases} 1 & y^*_{i,j} \geq 0 \\ 0 & y^*_{i,j} < 0 \end{cases}$$

$$y^*_{i,j} = \mathbf{x}'_{i,j}\boldsymbol{\beta} + a_i + b_j + u_{|i,j|}$$

$$\begin{pmatrix} a_i \\ b_i \end{pmatrix} \sim N\left\{ \begin{pmatrix} 0 \\ 0 \end{pmatrix}, \begin{pmatrix} \sigma_a^2 & \\ \sigma_{ab} & \sigma_b^2 \end{pmatrix} \right\}, \qquad \rho_{ab} = \frac{\sigma_{ab}}{\sqrt{\sigma_a^2}\sqrt{\sigma_b^2}}$$

$$u_{|i,j|} \sim N(0, \sigma_u^2), \qquad \rho_{uu} = \sigma_u^2/(\sigma_u^2 + 1)$$

$$p_a = \frac{\sigma_a^2}{\sigma_a^2 + \sigma_b^2 + \sigma_u^2 + 1}$$

$$p_b = \frac{\sigma_b^2}{\sigma_a^2 + \sigma_b^2 + \sigma_u^2 + 1}$$

$$p_u = \frac{\sigma_u^2 + 1}{\sigma_a^2 + \sigma_b^2 + \sigma_u^2 + 1}$$

Supplemental Figure 1. Predictions of Model 5 showing the effect of *distance* on the probability of helping. Model predictions assume a *degree of relatedness* of 0.125, and other predictors are held constant at their means or reference values. Shaded areas depict the 89th percentile confidence intervals around the model predictions.

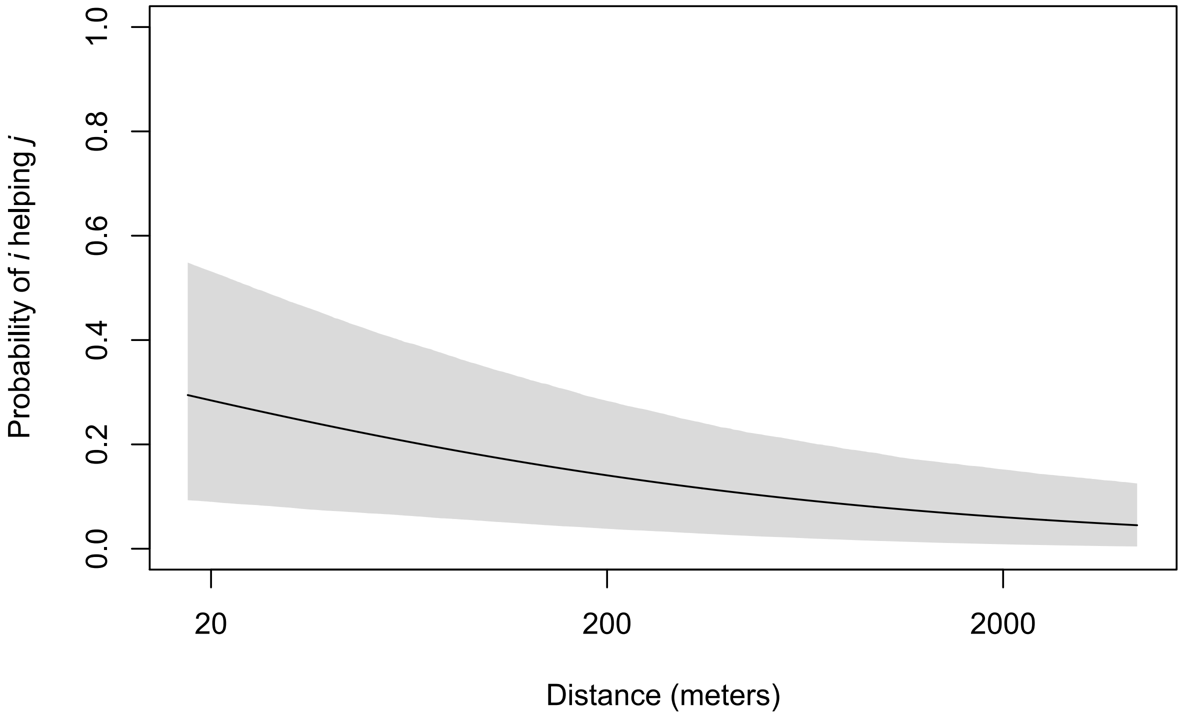

Supplemental Figure 2. Predictions of Model 5 showing the interacting effects of the *sex* of individual *i* and *j*. Females are noted as “F” and males as “M,” and the four predictions show the different combinations of male and female providers and recipients of aid, respectively (the provider of aid is listed first in the labels on the x-axis). Model predictions assume a *degree of relatedness* of 0.125, and other predictors are held constant at their means or reference values. Confidence bars depict the 89th percentile confidence intervals around the model predictions.

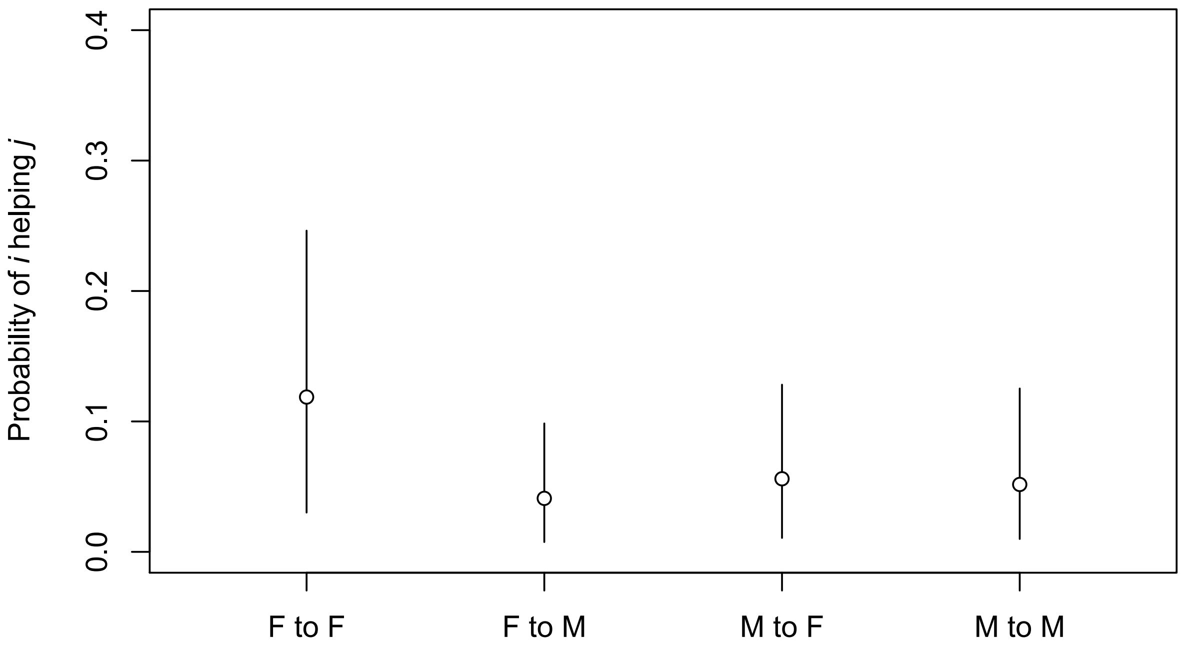

Supplemental Figure 3. Predictions of Model 5 showing the interacting effects of the ages of the provider (left) and recipient (right) of assistance. Model predictions assume a *degree of relatedness* of 0.125, and other predictors are held constant at their means or reference values. Shaded areas depict the 89th percentile confidence intervals around the model predictions. As noted in the text, these models are based on nominations by individual *j* and subject to latent heterogeneity in terms of how these individuals interpreted the question.

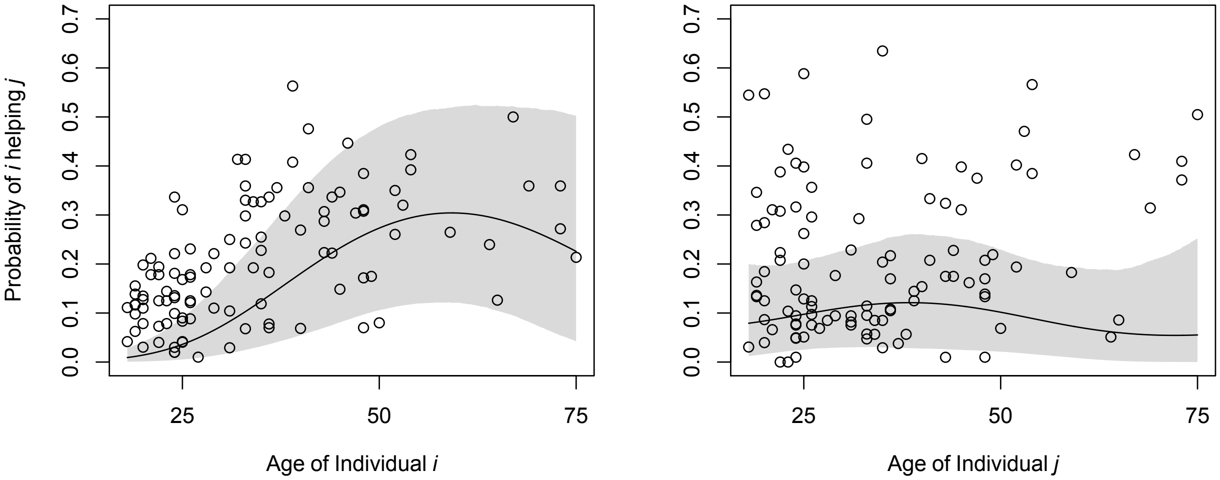

Supplemental Table 1. Model variances (σ^2), correlations (ρ), and variance partition coefficients (*p*) for the donor-oriented models. Reported quantities are the posterior means (standard deviations in parentheses).

	Parameter	Model 0'	Model 1'	Model 2'	Model 3'	Model 4'	Model 5'	Model 6'
σ_{a2}^2	House *k* variance		0.13 (0.14)	0.14 (0.14)	0.14 (0.15)	0.12 (0.14)	0.15 (0.15)	0.14 (0.15)
σ_{b2}^2	House *l* variance		0.21 (0.15)	0.28 (0.15)	0.30 (0.16)	0.29 (0.15)	0.29 (0.15)	0.31 (0.15)
σ_{a1}^2	Actor *i* variance	1.14 (0.19)	1.48 (0.29)	1.34 (0.29)	1.41 (0.30)	1.40 (0.31)	1.36 (0.29)	1.40 (0.30)
σ_{b1}^2	Partner *j* variance	0.86 (0.14)	0.84 (0.18)	0.37 (0.10)	0.38 (0.10)	0.38 (0.10)	0.37 (0.09)	0.36 (0.10)
σ_c^2	Actor-group variance		0.72 (0.12)	0.72 (0.14)	0.75 (0.14)	0.75 (0.14)	0.72 (0.13)	0.75 (0.14)
σ_d^2	Partner-group variance		0.47 (0.09)	0.46 (0.10)	0.47 (0.10)	0.48 (0.10)	0.45 (0.09)	0.48 (0.10)
σ_h^2	Group-level dyad variance		1.82 (0.27)	0.47 (0.11)	0.49 (0.12)	0.49 (0.12)	0.43 (0.10)	0.39 (0.11)
σ_u^2	Dyadic variance $\|ij\|$	1.78 (0.16)	0.30 (0.12)	0.30 (0.14)	0.34 (0.14)	0.33 (0.15)	0.31 (0.12)	0.35 (0.15)
ρ_{a2b2}	Group-level generalized reciprocity		0.23 (0.32)	0.20 (0.29)	0.20 (0.30)	0.15 (0.31)	0.23 (0.29)	0.21 (0.31)
ρ_{a1b1}	Actor-level generalized reciprocity	0.46 (0.08)	0.38 (0.10)	0.22 (0.12)	0.23 (0.12)	0.23 (0.12)	0.22 (0.12)	0.21 (0.12)
ρ_{cd}	Actor-group reciprocity		0.63 (0.08)	0.55 (0.09)	0.54 (0.09)	0.54 (0.09)	0.55 (0.08)	0.54 (0.08)
ρ_{hh}	Group-level dyadic reciprocity		0.94 (0.02)	0.81 (0.08)	0.81 (0.08)	0.81 (0.09)	0.79 (0.09)	0.78 (0.10)
ρ_{uu}	Actor-level dyadic reciprocity	0.64 (0.02)	0.22 (0.07)	0.22 (0.08)	0.25 (0.08)	0.24 (0.08)	0.23 (0.07)	0.25 (0.08)
p_{a2}	House *k* VPC		0.02 (0.02)	0.03 (0.03)	0.03 (0.03)	0.02 (0.02)	0.03 (0.03)	0.03 (0.03)
p_{b2}	House *l* VPC		0.03 (0.02)	0.06 (0.03)	0.06 (0.03)	0.06 (0.03)	0.06 (0.03)	0.06 (0.03)
p_{a1}	Actor *i* VPC	0.24 (0.03)	0.21 (0.03)	0.26 (0.04)	0.27 (0.04)	0.27 (0.04)	0.27 (0.04)	0.27 (0.04)
p_{b1}	Partner *j* VPC	0.18 (0.02)	0.12 (0.02)	0.07 (0.02)	0.07 (0.02)	0.07 (0.02)	0.07 (0.02)	0.07 (0.02)
p_c	Actor-group VPC		0.10 (0.01)	0.14 (0.02)	0.14 (0.02)	0.14 (0.02)	0.14 (0.02)	0.14 (0.02)
p_d	Partner-group VPC		0.07 (0.01)	0.09 (0.01)	0.09 (0.01)	0.09 (0.01)	0.09 (0.01)	0.09 (0.01)
p_h	Group-level dyad VPC		0.26 (0.02)	0.09 (0.02)	0.09 (0.02)	0.09 (0.02)	0.08 (0.02)	0.08 (0.02)
p_u	Dyadic VPC	0.58 (0.03)	0.19 (0.02)	0.26 (0.02)	0.26 (0.02)	0.26 (0.02)	0.26 (0.02)	0.26 (0.02)

Supplemental Table 2. Estimates for fixed effect parameters from the donor-oriented models. Reported quantities are the posterior means (standard deviations in parentheses). Interaction terms are denoted by an asterisk.

	Parameter	Model 0'	Model 1'	Model 2'	Model 3'	Model 4'	Model 5'	Model 6'
β_0	Intercept	–1.55 (0.17)	–1.70 (0.23)	–.12 (.61)	–.03 (.61)	–.13 (.62)	–.12 (.60)	–.10 (.60)
β_1	Male (Actor *i*)			–.91 (.27)	–.93 (.27)	–.92 (.27)	–.92 (.26)	–.96 (.27)
β_2	Male (Partner *j*)			–.28 (.15)	–.29 (.16)	–.29 (.15)	–.29 (.15)	–.32 (.15)
β_3	Male * Male (Actor-partner)			.47 (.10)	.48 (.10)	.47 (.10)	.46 (.10)	.47 (.10)
β_4	Age (Actor *i*)			.38 (.17)	.40 (.17)	.38 (.18)	.39 (.16)	.40 (.18)
β_5	Age^2 (Actor *i*)			–.07 (.11)	–.08 (.11)	–.07 (.11)	–.08 (.11)	–.07 (.11)
β_6	Age (Partner *j*)			.67 (.11)	.68 (.11)	.68 (.11)	.67 (.10)	.69 (.11)
β_7	Age^2 (Partner *j*)			–.08 (.07)	–.08 (.07)	–.08 (.07)	–.08 (.07)	–.08 (.07)
β_8	BMI (Actor *i*)			–.11 (.14)	–.12 (.14)	–.11 (.14)	–.12 (.14)	–.11 (.14)
β_9	BMI (Partner *j*)			.00 (.08)	–.01 (.09)	–.01 (.08)	–.02 (.08)	–.01 (.08)
β_{10}	BMI * BMI (Actor-partner)			–.01 (.03)	–.01 (.03)	–.01 (.03)	–.01 (.03)	–.01 (.03)
β_{11}	Skin color (Actor *i*)			–.08 (.13)	–.09 (.14)	–.09 (.13)	–.08 (.13)	–.10 (.14)
β_{12}	Skin color (Partner *j*)			–.10 (.08)	–.10 (.08)	–.10 (.09)	–.10 (.08)	–.11 (.08)
β_{13}	Skin color (Actor-partner)			–.03 (.03)	–.03 (.03)	–.03 (.03)	–.03 (.03)	–.03 (.03)
β_{14}	Godparental relationship			1.09 (.17)	1.12 (.17)	1.12 (.17)	1.09 (.16)	1.12 (.17)
β_{15}	Wealth (Actor house *k*)			.02 (.15)	.06 (.16)	.01 (.16)	.03 (.15)	.03 (.15)
β_{16}	Wealth (Partner house *l*)			–.06 (.12)	–.02 (.13)	–.06 (.13)	–.05 (.12)	–.05 (.12)
β_{17}	Distance			–.27 (.09)	–.28 (.09)	–.28 (.09)	–.26 (.08)	–.26 (.08)
β_{18}	Degree of relatedness			6.97 (.69)	6.47 (.85)	7.09 (.70)	7.06 (.67)	4.27 (1.16)
β_{19}	Affinal relatedness			3.09 (.41)	3.14 (.42)	3.14 (.42)	3.14 (.41)	1.58 (.85)
β_{20}	Average relatedness			.66 (.09)	.66 (.09)	.72 (.13)	.68 (.08)	.65 (.09)
β_{21}	Wealth * Wealth (House *k* and House *l*)				.02 (.05)	.00 (.06)		
β_{22}	Degree of relatedness * Wealth (House *k*)				–.48 (.46)			
β_{23}	Degree of relatedness * Wealth (House *l*)				–.16 (.45)			
β_{24}	Degree of relatedness * Wealth * Wealth				.14 (.36)			
β_{25}	Average relatedness * Wealth (House *k*)					.07 (.07)		
β_{26}	Average relatedness * Wealth (House *l*)					.04 (.07)		
β_{27}	Average relatedness * Wealth * Wealth					.05 (.05)		
β_{28}	Infidelity ties						–1.75 (.46)	–1.77 (.47)
β_{29}	Degree of relatedness * Average relatedness							1.75 (.62)
β_{30}	Affinal relatedness * Average relatedness							.99 (.48)
